# Occupational safety and health guidelines in relation to COVID‐19 risk, death risk, and case‐fatality proportion: An international, ecological study

**DOI:** 10.1002/hsr2.539

**Published:** 2022-03-14

**Authors:** Morgan Roberts, Steven M. Thygerson, John D. Beard, Camille Clark, Emma Montague

**Affiliations:** ^1^ Department of Public Health Brigham Young University Provo Utah USA

**Keywords:** adherence, coronavirus, COVID‐19, ecological, guidance, international, occupational, pandemic, workers

## Abstract

**Background:**

Coronavirus disease 2019 (COVID‐19) began in 2019 with several unknown factors. The World Health Organization (WHO) subsequently developed COVID‐19 occupational safety and health (OSH) guidelines to reduce occupational COVID‐19 transmission. Many countries also developed their own COVID‐19 OSH guidelines, but whether these guidelines included WHO's guidelines and whether including WHO's guidelines in countries' COVID‐19 OSH guidelines reduced COVID‐19 transmission is unknown.

**Objectives:**

The objectives of our study were to (1) compare the COVID‐19 OSH guidelines of several countries to WHO's OSH guidelines, (2) estimate associations between characteristics of countries and their OSH guidelines and the number of WHO's OSH guidelines included in countries' OSH guidelines, and (3) estimate associations between WHO's OSH guidelines included in countries' OSH guidelines and COVID‐19 risk, death risk, and case‐fatality proportion.

**Methods:**

This study represents international, ecological research of 36 countries from all six world health regions. Countries' COVID‐19 OSH guidelines were compared with WHO's OSH guidelines. Linear regression models adjusted for potential confounders were used to estimate associations of interest.

**Results:**

The median number of WHO's 15 COVID‐19 OSH guidelines included in countries' COVID‐19 OSH guidelines was eight. Countries' COVID‐19 OSH guidelines focused on workers included significantly more of WHO's COVID‐19 OSH guidelines than countries' COVID‐19 OSH guidelines focused on general populations. Including “provide personal protective equipment for workers” and “create workplace policy for wearing personal protective equipment” in countries' COVID‐19 OSH guidelines were significantly related to decreased COVID‐19 risk, death risk, and/or case‐fatality proportion.

**Conclusions:**

Countries' COVID‐19 OSH guidelines should include WHO's guidelines, focus on workers, and include “provide personal protective equipment for workers” and “create workplace policy for wearing personal protective equipment.”

## 1 INTRODUCTION

Coronavirus disease 2019 (COVID‐19) began in 2019 with several unknown factors. It was first discovered in Wuhan, China, and epidemiologists believe it likely started at an animal‐based food market.^1^ Severe acute respiratory syndrome (SARS) coronavirus 2 (SARS‐CoV‐2) is the virus that causes COVID‐19.^1^

The first known case of COVID‐19 was reported on December 1, 2019.^1^ However, it was not confirmed as a new strand of coronavirus for 38 days after that. This strand is very similar to both the severe acute respiratory syndrome (SARS) coronavirus (SARS‐CoV), which causes SARS, and the Middle East respiratory syndrome (MERS) coronavirus (MERS‐CoV), which causes MERS. SARS‐CoV‐2 mainly affected the respiratory system, has a similar incubation time, and transmits from human to human like other coronaviruses.^2^ SARS‐CoV‐2 seemed to cause symptoms of pneumonia, but at that time medical personnel did not know the incubation time, transmission pathway(s), or the original origin.^1^

On January 12, 2020, the World Health Organization (WHO) issued its first world travel warning and guidelines regarding how countries can prepare for this novel coronavirus. After this warning, COVID‐19 continued to spread to many other countries thereby initiating a pandemic that has altered the current state of the world.^1^ Differing information from Ministries of Health, other government entities, and organizations caused an “infodemic” where “news” traveled fast about the cause, prevention, and spread of COVID‐19. This caused confusion and a lack of trust in many countries regarding the methods that are effective in preventing COVID‐19.^3^

WHO has played a large role in the COVID‐19 pandemic response. In this worldwide crisis, WHO has issued several guidelines and warnings to limit the spread of COVID‐19 among the general public.^4^ In May 2020, WHO published COVID‐19 occupational safety and health (OSH) guidelines in a document titled “Considerations for public health and social measures in the workplace in the context of COVID‐19.”^5^ This document included different prevention and control guidelines for workers and workplaces to limit COVID‐19 spread. Additionally, WHO encouraged all countries to publish COVID‐19 OSH guidelines that aligned with WHO's guidelines.^5^

Many countries have released OSH guidelines as COVID‐19 has continued to spread worldwide. These guidelines range in depth from infographics containing sanitation measures to full articles regarding how employers should keep their workplaces best‐equipped to prevent COVID‐19. The purposes of our study were to (1) compare the COVID‐19 OSH guidelines of several countries to WHO's OSH guidelines, (2) estimate associations between the characteristics of countries and their OSH guidelines and the number of WHO's OSH guidelines included in countries' OSH guidelines, and (3) estimate associations between WHO's OSH guidelines included in countries' OSH guidelines and COVID‐19 risk, death risk, and case‐fatality proportion.

## 2 METHODS

### 2.1 Study design and sample

An ecological (i.e., the unit of analysis was the country) study design was used. Six countries from each of the WHO's six world health regions were selected (i.e., 36 countries total): Africa, Americas, Eastern Mediterranean, Europe, South‐East, and Western Pacific.^6^ Although a convenience sample was used, the study tried to include countries with a broad range of populations, densities, gross domestic products (GDP) per capita, COVID‐19 risk, and so on. The 36 countries included in our study were Afghanistan, Argentina, Australia, Botswana, Canada, China, Estonia, Ethiopia, France, Germany, Greece, India, Indonesia, Italy, Jamaica, Japan, Jordan, Kenya, Luxembourg, Maldives, Mexico, Nepal, New Zealand, Nigeria, Pakistan, Singapore, Sri Lanka, Syrian Arab Republic, Thailand, the United Kingdom, Turks and Caicos Islands, Uganda, United Arab Emirates, United States of America, Viet Nam, and Zambia. The 36 countries had an estimated combined midyear population in 2020 of 4,955,536,167, which was 65% of the estimated global midyear population in 2020.^7^

### 2.2 Data

Data were obtained for COVID‐19 cases and deaths through December 2, 2020, from WHO^8^ because December 2, 2020, was the date the United Kingdom became the first country to approve a COVID‐19 vaccine based on phase III clinical trial data.^9^ Data were obtained for midyear population, density, and age for the year 2020 from the United States of America's (US) Census Bureau.^7^ Data for GDP per capita in 2019 US dollars came from The World Bank Group.^10^ Data for WHO's COVID‐19 OSH guidelines came from WHO.^5^ Several sources provided the data for the 36 countries' COVID‐19 OSH guidelines.^11^, ^12^, ^13^, ^14^, ^15^, ^16^, ^17^, ^18^, ^19^, ^20^, ^21^, ^22^, ^23^, ^24^, ^25^, ^26^, ^27^, ^28^, ^29^, ^30^, ^31^, ^32^, ^33^, ^34^, ^35^, ^36^, ^37^, ^38^, ^39^, ^40^, ^41^, ^42^, ^43^, ^44^, ^45^, ^46^ Only COVID‐19 OSH guidelines that were available in English were used, but not every country had guidelines in English available from their Ministry of Health, Ministry of Labor, or other government entities. Therefore, information from other sources, such as from legal websites that cited government guidelines, was used as needed. The following country's guidance documents were translated to English: Argentina, Estonia, and Luxembourg.

### 2.3 Content analysis

A content analysis was conducted by comparing the 36 countries' COVID‐19 OSH guidelines to WHO's OSH guidelines. WHO's COVID‐19 OSH guidelines were considered as the gold standard comparison because of our study's international focus. These guidelines apply to workers in nonhealthcare settings. Fifteen specific guidelines in WHO's COVID‐19 OSH guidelines were identified:

1. Determine workplace level of risk (through a risk assessment).
2. Decide on the ability to reopen according to risks.
3. Encourage regular handwashing.
4. Provide handwashing or sanitation stations in the workplace.
5. Provide personal protective equipment (PPE) for all workers.
6. Create a workplace policy for wearing PPE at work.
7. Require 1 m of physical distancing.
8. Rearrange the workplace to include physical barriers to promote physical distancing (such as glass, queue management, etc.).
9. Stagger shifts or have employees telework when possible.
10. Cancel/postpone work travel.
11. Disinfect workplace regularly (especially high‐touch surfaces).
12. Create an environment with continuous COVID‐19 education in the workplace.
13. Require sick/symptomatic workers to stay home and quarantine, as well as implement protocols, to limit their exposure as they leave the workplace if symptoms onset during work hours.
14. Increase ventilation rate by natural or artificial means (avoid recirculation, especially for medium‐high risk workplaces).
15. Create a workplace plan of action for the prevention of COVID‐19.^5^

Two raters were trained to review each countries' COVID‐19 OSH guidelines and determine whether (i.e., “yes” or “no”) they included WHO's 15 COVID‐19 OSH guidelines. If the two reviewers disagreed, then a third trained rater review the discrepant guidelines and make the tiebreaking decision.

### 2.4 Statistical analyses

SAS version 9.4 (SAS Institute Inc.) was used to conduct all statistical analyses, and the country was used as the unit of analysis. Percent agreement and *κ*, and 95% confidence intervals (CI) for *κ*, were calculated to estimate the agreement between the two raters' determinations of whether countries' COVID‐19 OSH guidelines included WHO's OSH guidelines.^5^

The US Census Bureau's information on the age of population for each country was available as the number of people in 5‐year age categories: 0–4, 5–9,…, 90–94, 95–99, >99.^7^ To calculate the average age of population for each country, the midpoints of each age category were multiplied by the number of people in that age category, the products were summed, and the sums were divided by the total population. COVID‐19 risk per 1,000,000 people, COVID‐19 death risk per 1,000,000 people, and COVID‐19 case‐fatality proportion were calculated for each country using WHO's information on COVID‐19 cases and deaths, the US Census Bureau's information on population, and the following formulas^7^, ^8^:

$$\begin{array}{l} \text{COVID‐19 risk per 1,000,000 people}\\ \;=\;\left(\frac{\mathrm{new}\;\mathrm{cases}}{\mathrm{population}\;\mathrm{at}\;\mathrm{risk}}\right)\;\text{× 1,000,000,}\\ \text{COVID‐19 death risk per 1,000,000 people}\\ \;=\;\left(\frac{\mathrm{new}\;\mathrm{death}}{\mathrm{population}\;\mathrm{at}\;\mathrm{risk}}\right)\;\text{× 1,000,000,}\\ \text{COVID‐19 case‐fatality proportion}\\ \;=\;\left(\frac{\mathrm{new}\;\mathrm{deaths}}{\mathrm{new}\;\mathrm{cases}}\right)\;\text{× 100.} \end{array}$$

For continuous characteristics of countries and countries' COVID‐19 OSH guidelines included in our study, the mean, standard deviation, minimum, first quartile, median, third quartile, and maximum were calculated. For categorical characteristics of countries and countries' COVID‐19 OSH guidelines included in our study, the frequency and percentage were calculated.

A number of WHO's OSH guidelines were normally distributed, so simple linear regression models were used to estimate unadjusted means or changes in means, 95% CI, and *p*‐values for associations between characteristics of countries and countries' COVID‐19 OSH guidelines (i.e., exposure variable) and number of WHO's OSH guidelines included in countries' COVID‐19 OSH guidelines (i.e., outcome variable). The means for categorical characteristics of countries and countries' COVID‐19 OSH guidelines and changes in means (i.e., regression coefficients) were used for continuous characteristics of countries and countries' COVID‐19 OSH guidelines. Several versions (e.g., linear and categorical) of density, the average age of the population, and GDP per capita were considered for these analyses and the versions with the lowest values of the Akaike Information Criteria (AIC) were used.^47^, ^48^

Multivariable linear regression models were used to estimate adjusted geometric means (GM) or geometric mean ratios (GMR), 95% CI, and *p*‐values for associations between each of WHO's OSH guidelines and the number of WHO's OSH guidelines (i.e., exposure variable) and (1) COVID‐19 risk, (2) COVID‐19 death risk, and (3) COVID‐19 case‐fatality proportion (i.e., outcome variable) separately. All three outcomes (i.e., COVID‐19 risk, death risk, and case‐fatality proportion) were right skewed, so GMs or GMRs were used for individual WHO OSH guidelines and number of WHO's OSH guidelines. The multivariable linear regression models were adjusted for potential confounders selected from world health region, density, average age of population, and GDP per capita. Several versions (e.g., linear, categorical, and restricted quadratic regression splines) of density, average age of the population, and GDP per capita were considered for these analyses and the versions with the lowest values of the AIC were used.^47^, ^48^ When restricted quadratic regression splines were considered, versions of density, average age of the population, and GDP per capita that were centered at the mean were used. Several versions (e.g., linear and categorical) of the number of WHO's OSH guidelines were considered and the version with the lowest value of the AIC was used.^47^, ^48^ The change in mean squared error approach was used as described by Greenland et al.,^49^ which considers the bias‐variance tradeoff, to select which of the four potential confounders to adjust for in the multivariable linear regression models. The best balance of bias and variance for COVID‐19 risk was adjusting for world health region, density (linear), and GDP per capita (linear). For COVID‐19 death risk, the best balance of bias and variance was adjusting for world health region, density (3.3–150.0, 150.1–300.0, 300.1–8755.9), average age of the population (restricted quadratic regression spline with six equally spaced knots based on the entire distribution), and GDP per capita (linear). The best balance of bias and variance for COVID‐19 case‐fatality proportion was adjusting for world health region, density (linear), average age of the population (restricted quadratic regression spline with seven equally spaced knots based on the entire distribution), and GDP per capita (linear). All GMs, GMRs, and 95% CI were scaled to 1,000,000 people (COVID‐19 risk and death risk) or percentage (COVID‐19 case‐fatality proportion).

To determine whether associations between each of WHO's OSH guidelines and the three outcomes (i.e., COVID‐19 risk, death risk, and case‐fatality proportion) were confounded by the number of WHO's OSH guidelines included in countries' COVID‐19 OSH guidelines, the aforementioned multivariable linear regression model analyses were repeated with the number of WHO's OSH guidelines included in the models.

## 3 RESULTS

The overall percent agreement between the two raters' determinations of whether country documents included WHO's OSH guidelines was 81% (*κ* = 0.62; 95% CI: 0.56, 0.69; Table S1). However, percent agreement for WHO's 15 individual OSH guidelines ranged from 61% (*κ* = 0.28; 95% CI: 0.02, 0.53) for increased ventilation rate by natural or artificial means (avoid recirculation), especially for medium‐high risk workplaces, to 92% (*κ* = 0.83; 95% CI: 0.65, 1.00) for create workplace policy for wearing PPE.

For the 36 included countries, the median population was 40,473,526, the median density was 128.45 people/km^2^, and the median average age of the population was 34.20 years (Table 1). The median GDP per capita was 9934.61 in 2019 US dollars, median total COVID‐19 cases were 88,918.50, and median COVID‐19 risk per 1,000,000 people was 5737.75. The median total COVID‐19 deaths were 1501.50, median COVID‐19 death risk per 1,000,000 people was 54.03, and median COVID‐19 case‐fatality proportion was 1.74%.

**Table 1:** Characteristics of countries included in our study of WHO's COVID‐19 OSH guidelines included in countries’ COVID‐19 OSH guidelines, 2020

	Countries^a^								
Characteristic	*N*	%	Mean	SD	Min	Q1	Median	Q3	Max
Total	36	100							
World health region									
Africa	6	17							
Americas	6	17							
Eastern Mediterranean	6	17							
Europe	6	17							
South‐East Asia	6	17							
Western Pacific	6	17							
Population			137,653,782	311,330,257	55,926	10,299,567	40,473,526	103,417,213	1,394,015,977
Density, people/km^2^			422.04	1,446.36	3.30	57.60	128.45	251.15	8,755.90
Average age of population, years			34.20	7.60	20.40	27.80	34.20	39.92	47.71
Gross domestic product per capita, 2019 US dollars			22,101.56	25,907.79	502.12	2167.00	9934.61	41,289.14	114,704.59
Total COVID‐19 cases^b^			967,259.83	2,678,594.57	748.00	15,357.00	88,918.50	472,228.50	13,385,755.00
COVID‐19 risk per 1,000,000 people^b^			10,917.76	13,645.05	13.68	1047.17	5737.75	15,280.33	55,186.26
Total COVID‐19 deaths^b^			22,090.69	52,195.45	6.00	163.50	1501.50	14,605.50	266,043.00
COVID‐19 death risk per 1,000,000 people^b^			210.03	306.89	0.35	14.59	54.03	248.12	903.18
COVID‐19 case‐fatality proportion,^b^ %			2.18	1.79	0.05	0.97	1.74	2.93	9.51

Abbreviations: COVID‐19, coronavirus disease 2019; Max, maximum; Min, minimum; OSH, occupational safety and health; Q1, first quartile; Q3, third quartile; SD, standard deviation; US, United States of America; WHO, World Health Organization.

^a^ The 36 countries included in our study were Afghanistan, Argentina, Australia, Botswana, Canada, China, Estonia, Ethiopia, France, Germany, Greece, India, Indonesia, Italy, Jamaica, Japan, Jordan, Kenya, Luxembourg, Maldives, Mexico, Nepal, New Zealand, Nigeria, Pakistan, Singapore, Sri Lanka, Syrian Arab Republic, Thailand, the United Kingdom, Turks and Caicos Islands, Uganda, United Arab Emirates, United States of America, Viet Nam, and Zambia.

^b^ As of December 2, 2020, which was the date the United Kingdom became the first country to approve a COVID‐19 vaccine based on Phase III clinical trial data.

The percentage of countries' COVID‐19 OSH guidelines that focused on workers was 67% and 53% of countries' COVID‐19 OSH guidelines originated from the Ministry of Health or Ministry of Labor (Table 2). The percentage of countries' COVID‐19 OSH guidelines that contained WHO's individual OSH guidelines ranged from 22% for deciding on the ability to reopen to 92% for sick/symptomatic workers stay home and quarantine (limited exposure and leaving workplace) with a median of 50% for continuous COVID‐19 education in the workplace. The median number of WHO's OSH guidelines included in countries' COVID‐19 OSH guidelines was eight.

**Table 2:** Characteristics of and WHO's COVID‐19 OSH guidelines included in countries’ COVID‐19 OSH guidelines, 2020

	Countries^a^								
Variable	*N*	%	Mean	SD	Min	Q1	Median	Q3	Max
Total	36	100							
Focus of COVID‐19 guidelines									
Workers	24	67							
General population	12	33							
Source of COVID‐19 guidelines									
Ministry of Health	17	47							
Ministry of Labor	2	6							
Other government entity	6	17							
WHO	3	8							
Legal website sourcing Ministry of Health	8	22							
WHO's COVID‐19 OSH guidelines									
Determine workplace level of risk	17	47							
Decide on the ability to reopen	8	22							
Encourage regular handwashing	31	86							
Provide hand‐washing or sanitation stations in the workplace	23	64							
Provide personal protective equipment for workers	16	44							
Create workplace policy for wearing personal protective equipment	23	64							
One meter of physical distancing required	23	64							
Rearrange workplace to include physical barriers to promote physical distancing	16	44							
Stagger shifts/telework when possible	20	56							
Cancel/postpone work travel	16	44							
Disinfect workplace regularly (especially high‐touch surfaces)	27	75							
Continuous COVID‐19 education in the workplace	18	50							
Sick/symptomatic workers stay home and quarantine (limited exposure as leaving workplace)	33	92							
Increase ventilation rate by natural or artificial means (avoid recirculation), especially for medium‐high risk workplaces	12	33							
Create a workplace plan of action for the prevention of COVID‐19	17	47							
Number of WHO's COVID‐19 OSH guidelines included in countries’ COVID‐19 OSH guidelines			8.33	3.76	1.00	5.00	8.00	11.50	15.00

Abbreviations: COVID‐19, coronavirus disease 2019; Max, maximum; Min, minimum; OSH, occupational safety and health; Q1, first quartile; Q3, third quartile; SD, standard deviation, WHO, World Health Organization.

^a^ The 36 countries included in our study were Afghanistan, Argentina, Australia, Botswana, Canada, China, Estonia, Ethiopia, France, Germany, Greece, India, Indonesia, Italy, Jamaica, Japan, Jordan, Kenya, Luxembourg, Maldives, Mexico, Nepal, New Zealand, Nigeria, Pakistan, Singapore, Sri Lanka, Syrian Arab Republic, Thailand, United Kingdom, Turks and Caicos Islands, Uganda, United Arab Emirates, United States of America, Viet Nam, and Zambia.

The only characteristic of countries and countries' COVID‐19 OSH guidelines that was significantly associated with the number of WHO's OSH guidelines included in countries' COVID‐19 OSH guidelines was the focus of COVID‐19 guidelines (*p* = 0.01; Table 3). The countries' COVID‐19 OSH guidelines focused on workers had a mean number of WHO's OSH guidelines of 9.50 (95% CI: 8.08, 10.92), whereas the countries' COVID‐19 OSH guidelines focused on general populations had a mean of 6 (95% CI: 4, 8).

**Table 3:** Characteristics of countries and countries' COVID‐19 OSH guidelines in relation to the number of WHO's COVID‐19 OSH guidelines included in countries' COVID‐19 OSH guidelines, 2020

	Number^a^ of WHO's COVID‐19 OSH guidelines included in countries' COVID‐19 OSH guidelines			
Variable	Mean^b^	95% CI^b^		*p* value^b^
World health region				
Africa	9.17	5.96	12.37	
Americas	9.50	6.30	12.70	
Eastern Mediterranean	6.67	3.46	9.87	
Europe	8.83	5.63	12.04	
South‐East Asia	9.17	5.96	12.37	
Western Pacific	6.67	3.46	9.87	0.63
Density, 100 people/km^2^	−0.03^c^	−0.11^c^	0.05^c^	0.48
Average age of population,^d^ years				
20.40–26.83	7.50	4.86	10.14	
26.84–33.02	10.57	7.75	13.39	
33.03–38.28	6.43	3.61	9.25	
38.29–41.42	7.71	4.90	10.53	
41.43–47.71	9.57	6.75	12.39	0.23
Gross domestic product per capita, 2019 US $10,000	−0.10^c^	−0.57^c^	0.37^c^	0.67
Focus of COVID‐19 guidelines				
Workers	9.50	8.08	10.92	
General population	6.00	4.00	8.00	0.01
Source of COVID‐19 guidelines				
Ministry of Health	7.82	5.91	9.73	
Ministry of Labor	12.00	6.43	17.57	
Other government entity	8.33	5.12	11.55	
WHO	8.00	3.46	12.54	
Legal website sourcing Ministry of Health	8.63	5.84	11.41	0.71

Abbreviations: CI, confidence interval; COVID‐19, coronavirus disease 2019; OSH, occupational safety and health; US, United States of America; WHO, World Health Organization.

^a^ Possible values were 0 to 15.

^b^ Estimated via simple linear regression models of the original (i.e., untransformed) values.

^c^ Regression coefficient and 95% CI (i.e., change in the mean number of WHO's COVID‐19 OSH guidelines included in countries' COVID‐19 OSH guidelines for a specified change in the independent variable).

^d^ Category boundaries set at quintiles of the distribution of the variable.

Only two of WHO's individual OSH guidelines were significantly associated with COVID‐19 risk per 1,000,000 people: (1) provide PPE for workers (No: GM = 4715.55; 95% CI: 2871.85, 7742.88; Yes: GM = 2049.21; 95% CI: 1170.42, 3587.81; *p* = 0.04) and (2) create workplace policy for wearing PPE (No: GM = 6180.55; 95% CI: 3255.16, 11,734.97; Yes: GM = 2266.38; 95% CI: 1426.31, 3601.24; *p* = 0.02; Table 4). Both guidelines were associated with lower COVID‐19 risk per 1,000,000 people. The number of WHO's OSH guidelines included in countries' COVID‐19 OSH guidelines was not significantly associated with COVID‐19 risk per 1,000,000 people (*p* = 0.77).

**Table 4:** WHO's COVID‐19 OSH guidelines included in countries' COVID‐19 OSH guidelines in relation to COVID‐19 risk per 1,000,000 people,^a^ 2020

	COVID‐19 risk per 1,000,000 people^a^			
WHO's COVID‐19 OSH guidelines	GM^b^	95% CI^b^		*p* value^b^
Determine workplace level of risk				
No	3242.35	1872.66	5613.83	
Yes	3271.05	1823.67	5867.13	0.98
Decide on the ability to reopen				
No	3438.91	2199.01	5377.94	
Yes	2688.60	1055.04	6851.47	0.66
Encourage regular handwashing				
No	1724.19	622.72	4773.90	
Yes	3607.42	2424.95	5366.49	0.19
Provide hand‐washing or sanitation stations in the workplace				
No	2990.21	1555.94	5746.59	
Yes	3416.33	2110.87	5529.16	0.76
Provide personal protective equipment for workers				
No	4715.55	2871.85	7742.88	
Yes	2049.21	1170.42	3587.81	0.04
Create workplace policy for wearing personal protective equipment				
No	6180.55	3255.16	11,734.97	
Yes	2266.38	1426.31	3601.24	0.02
One meter of physical distancing required				
No	2368.12	1203.56	4659.51	
Yes	3897.76	2390.64	6355.01	0.27
Rearrange workplace to include physical barriers to promote physical distancing				
No	3015.15	1805.96	5033.97	
Yes	3583.96	2014.71	6375.49	0.67
Stagger shifts/telework when possible				
No	3028.81	1638.45	5598.99	
Yes	3449.71	2009.33	5922.65	0.77
Cancel/Postpone work travel				
No	2605.75	1567.76	4330.97	
Yes	4301.15	2426.95	7622.68	0.21
Disinfect workplace regularly (especially high‐touch surfaces)				
No	3990.85	1669.23	9541.46	
Yes	3042.29	1926.89	4803.36	0.61
Continuous COVID‐19 education in the workplace				
No	3048.22	1666.20	5576.56	
Yes	3477.66	1900.93	6362.19	0.79
Sick/symptomatic workers stay home and quarantine (limited exposure as leaving workplace)				
No	944.26	228.56	3901.08	
Yes	3643.66	2489.79	5332.28	0.08
Increase ventilation rate by natural or artificial means (avoid recirculation), especially for medium‐high risk workplaces				
No	2871.08	1785.93	4615.59	
Yes	4187.06	2082.93	8416.74	0.40
Create a workplace plan of action for the prevention of COVID‐19				
No	2815.39	1662.57	4767.56	
Yes	3830.20	2190.82	6696.34	0.44
Number of WHO's COVID‐19 OSH guidelines included in countries’ COVID‐19 OSH guidelines	1.02^c^	0.91^c^	1.13^c^	0.77

Abbreviations: CI, confidence interval; COVID‐19, coronavirus disease 2019; GM, geometric mean; OSH, occupational safety and health; WHO, World Health Organization.

^a^ As of December 2, 2020, which was the date United Kingdom became the first country to approve a COVID‐19 vaccine based on Phase III clinical trial data.

^b^ Estimated via multivariable linear regression models of the natural logarithm transformed values adjusted for world health region (Africa, Americas, Eastern Mediterranean, Europe, South‐East Asia, Western Pacific), density (linear term), and gross domestic product per capita (linear term).

^c^ Exponentiated regression coefficient and 95% CI (i.e., GM COVID‐19 case risk ratio for a one number change in WHO's COVID‐19 OSH guidelines included in countries’ COVID‐19 OSH guidelines or exp(*β*) – 1 = percent change in GM COVID‐19 case risk for a one number change in WHO's COVID‐19 OSH guidelines included in countries’ COVID‐19 OSH guidelines).

Only three of WHO's individual OSH guidelines were significantly associated with COVID‐19 death risk per 1,000,000 people: (1) encourage regular handwashing (*p* = 0.003), (2) provide PPE for workers (*p* = 0.009), and (3) create workplace policy for wearing PPE (*p* < 0.0001; Table 5). Encourage regular handwashing was associated with higher COVID‐19 death risk per 1,000,000 people (No: GM = 9.97; 95% CI: 3.65, 27.27; Yes: GM = 47.42; 95% CI: 33.30, 67.54), whereas provide PPE for workers (No: GM = 64.66; 95% CI: 39.77, 105.12; Yes: GM = 25.79; 95% CI: 15.72, 42.34) and create workplace policy for wearing PPE (No: GM = 111.54; 95% CI: 64.12, 194.04; Yes: GM = 25.39; 95% CI: 17.33, 37.20) were associated with lower COVID‐19 death risk per 1,000,000 people. The number of WHO's OSH guidelines included in countries' COVID‐19 OSH guidelines was significantly associated with COVID‐19 death risk per 1,000,000 people (*p* = 0.006). Countries that had eight to nine of WHO's OSH guidelines included in their COVID‐19 OSH guidelines had the highest COVID‐19 death risk per 1,000,000 people (GM = 128.29; 95% CI: 65.98, 249.43).

**Table 5:** WHO's COVID‐19 OSH guidelines included in countries’ COVID‐19 OSH guidelines in relation to COVID‐19 death risk per 1,000,000 people,^a^ 2020

	COVID‐19 death risk per 1,000,000 people^a^			
WHO's COVID‐19 OSH guidelines	GM^b^	95% CI^b^		*p* value^b^
Determine workplace level of risk				
No	34.36	19.48	60.63	
Yes	49.78	27.85	88.98	0.40
Decide on the ability to reopen				
No	48.13	31.94	72.55	
Yes	21.66	9.26	50.65	0.10
Encourage regular handwashing				
No	9.97	3.65	27.27	
Yes	47.42	33.30	67.54	0.003
Provide hand‐washing or sanitation stations in the workplace				
No	40.36	20.62	78.99	
Yes	41.60	26.43	65.46	0.94
Provide personal protective equipment for workers				
No	64.66	39.77	105.12	
Yes	25.79	15.72	42.34	0.009
Create workplace policy for wearing personal protective equipment				
No	111.54	64.12	194.04	
Yes	25.39	17.33	37.20	<0.0001
One meter of physical distancing required				
No	30.69	15.85	59.42	
Yes	47.95	30.06	76.49	0.29
Rearrange workplace to include physical barriers to promote physical distancing				
No	43.73	26.20	73.00	
Yes	38.06	20.96	69.12	0.74
Stagger shifts/telework when possible				
No	44.20	21.23	92.01	
Yes	39.75	24.13	65.46	0.83
Cancel/postpone work travel				
No	36.40	21.15	62.63	
Yes	46.68	27.10	80.41	0.53
Disinfect workplace regularly (especially high‐touch surfaces)				
No	37.35	14.52	96.04	
Yes	42.41	26.88	66.93	0.82
Continuous COVID‐19 education in the workplace				
No	32.85	18.32	58.92	
Yes	53.41	28.30	100.80	0.32
Sick/symptomatic workers stay home and quarantine (limited exposure as leaving workplace)				
No	10.80	1.99	58.60	
Yes	50.33	32.35	78.30	0.11
Increase ventilation rate by natural or artificial means (avoid recirculation), especially for medium‐high risk workplaces				
No	40.30	24.96	65.05	
Yes	42.98	22.08	83.64	0.88
Create a workplace plan of action for the prevention of COVID‐19				
No	32.74	19.31	55.53	
Yes	52.51	30.52	90.33	0.23
Number of WHO's COVID‐19 OSH guidelines included in countries’ COVID‐19 OSH guidelines				
1–5	21.28	10.47	43.28	
6–7	26.49	12.01	58.42	
8–9	128.29	65.98	249.43	
10–15	33.95	19.10	60.36	0.006

Abbreviations: CI, confidence interval; COVID‐19, coronavirus disease 2019; GM, geometric mean; OSH, occupational safety and health; WHO, World Health Organization.

^a^ As of December 2, 2020, which was the date United Kingdom became the first country to approve a COVID‐19 vaccine based on Phase III clinical trial data.

^b^ Estimated via multivariable linear regression models of the natural logarithm transformed values adjusted for world health region (Africa, Americas, Eastern Mediterranean, Europe, South‐East Asia, Western Pacific), density (3.3–150.0, 150.1–300.0, 300.1–8755.9), average age of the population (restricted quadratic regression spline with six equally spaced knots based on the entire distribution at −9.97, −5.19, −1.14, 1.83, 4.80, and 7.97 years; average age of the population was centered at the mean of 34.20 years), and gross domestic product per capita (linear term).

Only two of WHO's individual OSH guidelines were significantly associated with COVID‐19 case‐fatality proportion: (1) determine workplace level of risk (*p* = 0.05) and (2) create workplace policy for wearing PPE (*p* = 0.05; Table 6). Determine workplace level of risk was associated with higher COVID‐19 case‐fatality proportion (No: GM = 1.28; 95% CI: 1.01, 1.61; Yes: GM = 1.90; 95% CI: 1.48, 2.45), whereas create workplace policy for wearing PPE was associated with lower COVID‐19 case‐fatality proportion (No: GM = 1.98; 95% CI: 1.49, 2.65; Yes: GM = 1.34; 95% CI: 1.09, 1.64). The number of WHO's OSH guidelines included in countries' COVID‐19 OSH guidelines was not significantly associated with COVID‐19 case‐fatality proportion (*p* = 0.73).

**Table 6:** WHO's COVID‐19 OSH guidelines included in countries’ COVID‐19 OSH guidelines in relation to COVID‐19 case‐fatality proportion (%),^a^ 2020

	COVID‐19 case‐fatality proportion (%)^a^			
WHO's COVID‐19 OSH guidelines	GM^b^	95% CI^b^		*p* value^b^
Determine workplace level of risk				
No	1.28	1.01	1.61	
Yes	1.90	1.48	2.45	0.05
Decide on the ability to reopen				
No	1.51	1.26	1.82	
Yes	1.65	1.10	2.47	0.73
Encourage regular handwashing				
No	1.11	0.68	1.80	
Yes	1.62	1.38	1.92	0.16
Provide hand‐washing or sanitation stations in the workplace				
No	1.64	1.24	2.18	
Yes	1.49	1.21	1.82	0.60
Provide personal protective equipment for workers				
No	1.57	1.25	1.96	
Yes	1.51	1.17	1.94	0.83
Create workplace policy for wearing personal protective equipment				
No	1.98	1.49	2.65	
Yes	1.34	1.09	1.64	0.05
One meter of physical distancing required				
No	1.21	0.91	1.61	
Yes	1.76	1.44	2.16	0.06
Rearrange workplace to include physical barriers to promote physical distancing				
No	1.61	1.27	2.03	
Yes	1.46	1.12	1.91	0.64
Stagger shifts/telework when possible				
No	1.70	1.28	2.26	
Yes	1.42	1.11	1.82	0.42
Cancel/postpone work travel				
No	1.30	1.04	1.64	
Yes	1.90	1.46	2.47	0.06
Disinfect workplace regularly (especially high‐touch surfaces)				
No	1.84	1.20	2.82	
Yes	1.45	1.19	1.78	0.39
Continuous COVID‐19 education in workplace				
No	1.41	1.08	1.84	
Yes	1.68	1.29	2.20	0.43
Sick/symptomatic workers stay home and quarantine (limited exposure as leaving workplace)				
No	1.89	0.90	3.97	
Yes	1.51	1.28	1.78	0.58
Increase ventilation rate by natural or artificial means (avoid recirculation), especially for medium‐high risk workplaces				
No	1.57	1.28	1.93	
Yes	1.48	1.08	2.02	0.77
Create a workplace plan of action for the prevention of COVID‐19				
No	1.52	1.20	1.93	
Yes	1.56	1.21	2.02	0.88
Number of WHO's COVID‐19 OSH guidelines included in countries’ COVID‐19 OSH guidelines	1.01^c^	0.96^c^	1.07^c^	0.73

Abbreviations: CI, confidence interval; COVID‐19, coronavirus disease 2019; GM, geometric mean; OSH, occupational safety and health; WHO, World Health Organization.

^a^ As of December 2, 2020, which was the date the United Kingdom became the first country to approve a COVID‐19 vaccine based on Phase III clinical trial data.

^b^ Estimated via multivariable linear regression models of the natural logarithm transformed values adjusted for world health region (Africa, Americas, Eastern Mediterranean, Europe, South‐East Asia, Western Pacific), density (linear term), average age of the population (restricted quadratic regression spline with seven equally spaced knots based on the entire distribution at −10.10, −7.26, −2.26, 0.00, 4.46, 5.93, and 9.15 years; average age of the population was centered at the mean of 34.20 years), and gross domestic product per capita (linear term).

^c^ Exponentiated regression coefficient and 95% CI (i.e., GM COVID‐19 case‐fatality proportion ratio for a one number change in WHO's COVID‐19 OSH guidelines included in countries’ COVID‐19 OSH guidelines or exp(*β*) – 1 = percent change in GM COVID‐19 case‐fatality proportion for a one number change in WHO's COVID‐19 OSH guidelines included in countries’ COVID‐19 OSH guidelines).

Repeating all multivariable linear regression model analyses shown in Tables 4, 5, 6 additionally adjusting for the number of WHO's COVID‐19 OSH guidelines included in countries' COVID‐19 OSH guidelines gave results that were qualitatively similar to the results of the multivariable linear regression models that did not adjust for the number of WHO's COVID‐19 OSH guidelines included in countries' COVID‐19 OSH guidelines (not shown).

## 4 DISCUSSION

In this study of associations between COVID‐19 OSH guidelines and COVID‐19 risk, death risk, and case‐fatality proportion, the median number of WHO's 15 OSH guidelines included in countries' COVID‐19 OSH guidelines was 8. Countries' COVID‐19 OSH guidelines focused on workers had a significantly higher number of WHO's OSH guidelines included in their COVID‐19 OSH guidelines compared with countries whose COVID‐19 OSH guidelines focused on general populations. Finally, including “provide personal protective equipment for workers” and “create workplace policy for wearing personal protective equipment” in countries' COVID‐19 OSH guidelines were significantly associated with decreased COVID‐19 risk, death risk, and/or case‐fatality proportion, whereas including “encourage regular handwashing” was significantly associated with increased COVID‐19 death risk and including “determine workplace level of risk” was significantly associated with increased COVID‐19 case‐fatality proportion.

Countries' COVID‐19 OSH guidelines focused on workers had a significantly higher number of WHO's OSH guidelines included in their COVID‐19 OSH guidelines compared with countries whose COVID‐19 OSH guidelines focused on general populations. Preventing exposure to bloodborne pathogens (BBP) is another example in which having guidelines focused on workers can help prevent the transmission of infectious diseases. For example, the University of Pittsburgh Medical Center (UPMC) in US shows data regarding how to prevent and treat diseases caused by BBP through a presentation to medical students.^50^ They also have a UPMC BBP Exposure Control Plan for all workers as well as specific plans for each individual job. The plans describe PPE and other safety equipment to prevent transmission of BBP. Another example is the Florida Department of Health in the United States has guidelines for individuals who work with BBP and their guidelines coincide with the standards included in the US Occupational Safety and Health Administration's FactSheet “Bloodborne Pathogens Standards”.^51^ Forming clear occupational regulations and guidelines for specific infectious diseases could help prevent the occupational spread of COVID‐19 and other infectious diseases in the future.

No associations were found between world health region, density, average age of the population, GDP per capita, or source of COVID‐19 guidelines and number of WHO's COVID‐19 OSH guidelines included in countries' COVID‐19 OSH guidelines. However, if the source of COVID‐19 guidelines was the Ministry of Labor, then the number of WHO's COVID‐19 OSH guidelines included in countries' COVID‐19 OSH guidelines was three to four guidelines higher than if the source of COVID‐19 guidelines was not the Ministry of Health, which may have been a practically significant finding even though it was not a statistically significant finding. Thus, the Ministry of Labor should be involved in determining and developing OSH guidelines for reducing and preventing transmission of COVID‐19 and other infectious diseases.

An important finding from our study was that when countries' COVID‐19 OSH guidelines included “create workplace policy for wearing personal protective equipment,” there was a significant decrease in COVID‐19 risk, death risk, and case‐fatality proportion. Similarly, when countries' COVID‐19 OSH guidelines included “provide personal protective equipment for workers,” there was a significant decrease in COVID‐19 risk and death risk. Other studies have also found PPE, particularly masks, can limit the spread of COVID‐19. For example, one study reported two hairstylists were unaware they had COVID‐19 and continued working for 10 days. There were 139 clients that came into the salon during this time, but all stylists wore masks as well as 98% of the clientele and no one developed symptoms of COVID‐19.^52^ Research also shows masks do not endanger individuals, although they may cause minor reactions such as headaches and some skin irritation.^53^, ^54^ Among surgeons there was only a slight blood oxygen decrease while they wore masks, but their oxygen levels stayed in a healthy range and some of the decreases could be due to stress or other factors.^55^, ^56^ Providing masks or other PPE for workers and creating a workplace policy for wearing PPE or masks should be primary OSH guidelines used to stop occupational COVID‐19 transmission. An obstacle to implementing wearing masks or other PPE in the workplace is providing PPE for each employee would cost employers more than not providing PPE for employees. Misunderstanding the effectiveness of PPE in reducing COVID‐19 transmission and improper wearing of PPE are other obstacles, so education, such as the US Centers for Disease Control and Prevention's (CDC) information about how to wear masks properly could increase workers' compliance with PPE wearing policies and proper use of PPE.^57^, ^58^

Handwashing is the best practice for stopping the spread of infectious diseases because people often touch their faces, eyes, and mouths. Germs on the hands can carry infectious diseases such as, but not limited to, salmonella, *E. Coli* O157, and norovirus. In addition, the US CDC found that 20%–30% of diarrheal sicknesses and respiratory infections can be prevented by regular handwashing.^59^ A meta‐analysis found that handwashing is effective for mitigating the spread of gastrointestinal illness.^60^ Two other studies found the combined effect of hand hygiene (i.e., using alcohol‐based hand sanitizer) and face mask wearing reduced incidence of influenza.^61^, ^62^ In our study, including “encourage regular handwashing” in countries' COVID‐19 OSH guidelines was significantly positively associated with COVID‐19 death risk, but it was not significantly associated with COVID‐19 risk or case‐fatality proportion, which may suggest this finding could be attributed to chance and may not be valid.

Studies have found that several countries and businesses have used different ways to determine the workplace level of risk. In Italy, factors such as how close workers were to each other, if the activity at work was dealing directly with SARS‐CoV‐2, and third‐party participants' activities were all integrated into a formula to identify workplace level of risk.^63^ Another study found that safety models like Anticipate, Recognize, Evaluate, Control, and Confirm (ARECC), which was initially used for chemical hazards in the workplace, could be adapted for use with COVID‐19.^64^ Including “determine workplace level of risk” in countries' COVID‐19 OSH guidelines was significantly positively associated with COVID‐19 case‐fatality proportion in our study, but it was not significantly associated with COVID‐19 risk or death risk, which again may suggest this finding could be attributed to chance and may not be valid.

None of WHO's other COVID‐19 OSH guidelines were significantly related to COVID‐19 risk, death risk, or case‐fatality proportion in our study. In addition, the number of WHO's COVID‐19 OSH guidelines in countries' COVID‐19 OSH guidelines was not significantly associated with COVID‐19 risk or case‐fatality proportion, but it was significantly associated with COVID‐19 death risk. However, the association between the number of WHO's COVID‐19 OSH guidelines in countries' COVID‐19 OSH guidelines and COVID‐19 death risk was nonlinear. A possible explanation could be countries may have had detailed COVID‐19 OSH guidelines that complied with WHO's COVID‐19 OSH guidelines, but workers or employers in those countries may not have followed, implemented or enforced the guidelines in their workplaces. In fact, a longitudinal cohort study about whether youth in Switzerland followed COVID‐19 guidelines found “Noncompliance was strongly associated with weaker feelings of moral obligation and low trust in authorities, but also with characteristics related to antisocial potential.”^65^ Culture or inadequate policy may also play a role in noncompliance with COVID‐19 OSH guidelines. For example, in Nepal, a motorcycle driver will wear a helmet because doing so is required by the law, but he or she will not put a helmet on his or her motorcycle passengers, such as a spouse, child, or baby, because there is no law that requires helmet use by passengers.^66^

The connection between the COVID‐19 OSH guidelines and deaths is maybe not direct as a majority of COVID‐19 deaths is among retired elderly people. However, the percentage of elderly or retirement people in many countries who still work full‐ or part‐time is increasing.^67^ Although the majority of deaths from COVID‐19 occur among elderly people, there is a sizeable percentage of deaths from COVID‐19 that occur among working age people. Additionally, workers may have elderly or retired people who live with them or they visit elderly or retired people frequently; therefore, following COVID‐19 OSH guidelines at work will help workers minimize community spread when they are not at work.

Strengths of our study include its novel, relevant research question, and international focus. This study also adjusted for confounders. Although only 36 countries were included in our study, those 36 countries had an estimated combined midyear population in 2020 of 4,955,536,167, which was 65% of the estimated global midyear population in 2020.^7^ The WHO's COVID‐19 OSH guidelines were used as the gold standard to which to compared the countries' COVID‐19 OSH guidelines. Data were obtained from reputable sources, including the WHO, the US Census Bureau, and The World Bank. To minimize exposure misclassification, the study had up to three trained raters review each countries' COVID‐19 OSH guidelines to determine whether they included WHO's 15 COVID‐19 OSH guidelines. Finally, there were no missing data.

Limitations of our study include there was likely confounding by unmeasured, unidentified confounders. For example, only OSH guidelines were considered, but general population guidelines may have been associated with OSH guidelines and COVID‐19 risk, death risk, and/or case‐fatality proportion. This study was limited to reviewing countries' COVID‐19 OSH guidelines that were available in English. A convenience sample was used, but the study tried to include countries with a broad range of populations, densities, GDP per capita, COVID‐19 risk, and so on. Associations between COVID‐19 OSH guidelines and COVID‐19 risk, death risk, and case‐fatality proportion were estimated, but workers or employers in those countries may not have followed, implemented, or enforced the guidelines in their workplaces. COVID‐19 cases and deaths were likely underreported. Finally, our results may have been susceptible to ecological fallacy if the country‐level results of our study do not apply to or exist at the individual level.^68^

## 5 CONCLUSION

There is room for improvement in countries' COVID‐19 OSH guidelines because the median number of WHO's 15 COVID‐19 OSH guidelines included in countries' COVID‐19 OSH guidelines was eight. Countries that focused their COVID‐19 OSH guidelines on workers included significantly more of WHO's COVID‐19 OSH guidelines than countries that focused their COVID‐19 OSH guidelines on general populations. Including “provide personal protective equipment for workers” and “create workplace policy for wearing personal protective equipment” in countries' COVID‐19 OSH guidelines were significantly related to decreased COVID‐19 risk, death risk, and/or case‐fatality proportion. Therefore, it is recommended countries look to world COVID‐19 OSH guidelines, such as those developed by WHO, and use them as a standard to follow. It is recommended that countries' COVID‐19 OSH guidelines focus on workers and that countries create COVID‐19 guidelines for workers, employers, and workplaces in addition to general populations. Countries' COVID‐19 OSH guidelines should include “provide personal protective equipment for workers” and “create workplace policy for wearing personal protective equipment.” Finally, it is recommended that further research at the individual level to determine whether workers' compliance with COVID‐19 OSH guidelines is associated with COVID‐19 risk, death risk, and/or case‐fatality proportion.

## CONFLICTS OF INTEREST

The authors declare no conflicts of interest.

## AUTHOR CONTRIBUTIONS

**Morgan Roberts:** Conceptualization, data curation, investigation, methodology, project administration, writing—original draft, writing—review & editing. **Steven M. Thygerson:** Conceptualization, funding acquisition, investigation, methodology, project administration, resources, supervision, visualization, writing—review & editing. **John Beard:** Data curation, formal analysis, investigation, methodology, software, supervision, writing—review & editing. **Camille Clark:** Data curation, formal analysis, methodology, writing—original draft, writing—review & editing. **Emma Montague:** Investigation, methodology, writing—original draft.

## Supporting information

Supplementary information.
