# Supplementary material for: Occupational safety and health guidelines in relation to COVID‐19 risk, death risk, and case‐fatality proportion: An international, ecological study
Source: Health Sci Rep. 2022 Mar 14;5(2):e539. doi: 10.1002/hsr2.539 (PMC8919368; doi:10.1002/hsr2.539)
Supplement: Supplementary file 1 — Supplementary information. [file HSR2-5-e539-s001.docx]

Supplemental Material

**Occupational safety and health guidelines in relation to COVID-19 risk, death risk, and case fatality proportion: an international, ecological, cross-sectional study**

Table S1. Agreement among raters regarding whether countries’ COVID-19 OSH guidelines included WHO's COVID-19 OSH guidelines, 2020.

| WHO's COVID-19 OSH Guidelines | Percent Agreement | κ | 95% CI | |
| --- | --- | --- | --- | --- |
| Determine workplace level of risk | 75.00 | 0.49 | 0.21 | 0.77 |
| Decide on ability to reopen | 88.89 | 0.78 | 0.57 | 0.98 |
| Encourage regular handwashing | 88.89 | 0.78 | 0.57 | 0.98 |
| Provide hand-washing or Sanitation Stations in the workplace | 72.22 | 0.38 | 0.06 | 0.69 |
| Provide personal protective equipment for workers | 75.00 | 0.44 | 0.14 | 0.73 |
| Create workplace policy for wearing personal protective equipment | 91.67 | 0.83 | 0.65 | 1.00 |
| One meter of physical distancing required | 86.11 | 0.68 | 0.43 | 0.93 |
| Rearrange workplace to include physical barriers to promote physical distancing | 88.89 | 0.61 | 0.28 | 0.94 |
| Stagger shifts/telework when possible | 86.11 | 0.70 | 0.46 | 0.94 |
| Cancel/Post-pone work travel | 83.33 | 0.66 | 0.43 | 0.89 |
| Disinfect workplace regularly (especially high-touch surfaces) | 88.89 | 0.76 | 0.54 | 0.98 |
| Continuous COVID-19 education in workplace | 69.44 | 0.39 | 0.15 | 0.63 |
| Sick/symptomatic workers stay home and quarantine (limited exposure as leaving workplace) | 86.11 | 0.46 | 0.07 | 0.86 |
| Increase ventilation rate by natural or artificial means (avoid re-circulation), especially for medium-high risk workplaces | 61.11 | 0.28 | 0.02 | 0.53 |
| Create a workplace plan of action for prevention of COVID-19 | 77.78 | 0.53 | 0.25 | 0.82 |
| Overall | 81.30 | 0.62 | 0.56 | 0.69 |

Abbreviations: CI, confidence interval; COVID-19, coronavirus disease 2019; OSH, occupational safety and health; WHO, World Health Organization.
